# Considérations nosographiques sur le délire d'infestation parasitaire à travers trois observations cliniques

**DOI:** 10.11604/pamj.2016.24.130.6512

**Published:** 2016-06-09

**Authors:** Nabil Berhili, Amine Bout, Hayat Hlal, Chadya Aarab, Rachid Aalouane, Ismail Rammouz

**Affiliations:** 1 Université Sidi Mohammed Ben Abdellah, Faculté de Médecine et de Pharmacie, Service de Psychiatrie, Centre Hospitalier Universitaire Hassan II, 30000 Fès, Maroc

**Keywords:** Syndrome d′Ekbom, délire d′infestation parasitaire, conviction inébranlable, Ekbom syndrome, delirium of parasitic infestation, unwavering conviction

## Abstract

Le syndrome d'Ekbom, ou délire d'infestation parasitaire, est une pathologie rare, caractérisée par la conviction inébranlable d'avoir une peau infestée d'insectes ou de parasites. Il s'agit d'un délire monothématique à mécanisme hallucinatoire qui touche typiquement les femmes d’âge avancé. Nous rapportons les cas de trois patients qui présentent un délire d'infestation parasitaire dans des contextes cliniques différents. Le premier patient souffre d'un délire d'infestation isolé correspondant à la forme décrite par Karl Ekbom. Le deuxième cas présente un délire d'infestation secondaire, s'inscrivant dans le cadre d'une leuco-encéphalopathie de type CADASIL. Enfin, le troisième patient se présente dans un tableau évocateur d'un épisode dépressif avec une caractéristique psychotique qui intègre le délire d'infestation. Ces trois vignettes cliniques illustrent parfaitement le caractère trans-nosographique de ce syndrome ainsi que les difficultés de prise en charge de ces patients, tant au niveau de l'alliance thérapeutique que sur le plan des choix en matière de traitement pharmacologiques.

## Abstract (en)

Ekbom syndrome or delusional parasitosis is a rare disease characterized by the unwavering conviction of having cutaneous infestation of insects or parasites. This is a monothematic delusion of hallucinatory origin that typically affects older women. We report the case of three patients with delusional parasitosis in different clinical settings. The first patient suffered from isolated delusional parasitosis corresponding to the condition described by Karl Ekbom. The second case suffered from secondary delusional parasitosis, occurring in the setting of leukoencephalopathy (CADASIL). Lastly, the third patient appeared in a state which was evocative of a depressive episode with psychotic symptom integrating delusional parasitosis. These three clinical vignettes perfectly illustrate the trans-nosographic dimension of this syndrome and the difficulties in treating these patients, both in terms of therapeutic alliance and of choice of pharmacological treatment.

## Introduction

Le délire d'infestation parasitaire, ou syndrome d'Ekbom, individualisé par le médecin éponyme en 1938, est une affection rare caractérisée par la conviction inébranlable d'avoir une peau infestée d'insectes ou de parasites. Ce délire monothématique, sous-tendu par des hallucinations tactiles ou visuelles, s'accompagne généralement d'un vécu anxieux important. Il n'est pas rare que le patient apporte des preuves de son infestation sous forme d'une boite contenant des « spécimens » (généralement des poils ou des squames) renvoyant ainsi au classique «signe de la boite d'allumettes» [1]. D'un point de vue étiologique, il faudra nuancer entre le délire d'infestation parasitaire primaire et le délire dit secondaire [2, 3]. Dans la forme primaire, décrite par Ekbom, les hallucinations sont limitées à la peau et les idées délirantes sont monothématiques. La forme secondaire du délire, imputable à une pathologie psychiatrique ou organique, serait la plus fréquente selon Trabert [2]. Afin de mieux appréhender la difficulté diagnostique de ce syndrome, nous allons présenter trois vignettes cliniques, illustrant trois contextes nosographiques différents, puis nous discuterons les moyens de prise en charge de ce trouble.

## Patient et observations

### Observation n°1

Le premier patient est un homme de 53 ans, un père de famille qui exerce le métier de maçon. Il ne présente pas d'antécédents pathologiques personnels ou familiaux particuliers. Il consulte en psychiatrie à l'occasion d'une décompensation dépressive, se disant épuisé par la lutte contre un insecte qui infeste son corps depuis presque sept ans. Se déplaçant exclusivement au niveau de son hémicorps droit, l'insecte se frayerait un chemin sous sa peau en la mordant, ce qui engendre une douleur et un prurit intenses. Il rapporte que le trouble a débuté de façon insidieuse et s'est aggravé progressivement jusqu’à atteindre son paroxysme au terme de quelques semaines d’évolution. La revue de la trajectoire du malade met en évidence d'innombrables consultations chez des médecins somaticiens, essentiellement des dermatologues ou des neurologues. Le trouble a entrainé chez lui une altération conséquente sur le plan professionnel puisqu'il s'est retrouvé sans emploi depuis un an. Cependant le patient garde un assez bon fonctionnement familial et social. C'est d'ailleurs sur le conseil d'un ami qu'il consulte en psychiatrie, précisant bien qu'il cherche à soigner sa dépression et réfutant tout droit du psychiatre à traiter son problème « somatique ».

**Figure 1:**
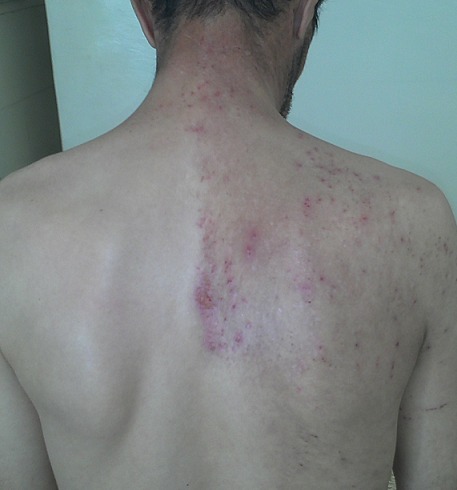
Une photo montrant l'ensemble des lésions que le patient s'est auto-infligé au niveau du dos l’épaule et la nuque afin d'extraire « l'insecte »

La première évaluation clinique avait permis de découvrir un patient très anxieux, avec une pensée qui demeurait centrée sur une idée délirante unique: « l'insecte qui infeste son corps ». Le patient affirme pourvoir sentir ses mouvements sous son doigt quand il tente de le bloquer et dit même être capable de le déplacer sous sa peau en lui faisant emprunter de longs trajets le long de son dos, en passant par sa nuque jusqu'au visage. Il se déshabille spontanément pour apporter les preuves au médecin, pointant du doigt des endroits correspondant à des lésions de grattages avec des petites cicatrices qu'il attribue aux points d'introduction d'une aiguille dont il se serait aidé pour tenter d'extraire le fameux insecte (Figure 1). Il n'hésite pas à demander au médecin psychiatre de toucher lui même ses endroits, afin qu'il puisse vérifier la véracité de ses dires et sentir les mouvements de l'insecte. D'ailleurs il exprime sa révolte contre l'impuissance voire l'incompétence de certains praticiens et tolère très mal l'incrédulité dont certains ont fait preuve. Par ailleurs, l’évaluation a permis de retenir un syndrome dépressif d'apparition récente, sans idées suicidaires, de ruine ou d'incurabilité comme en témoigne d'ailleurs la perpétuelle quête de soins du patient. Sur le plan perceptif, en dehors de la composante tactile ou cénesthésique qui sous-tend sa conviction délirante on n'a pas retrouvé d'autres éléments hallucinatoires. Il en va de même pour le mécanisme interprétatif, quasi-absent du discours du patient. Les entretiens suivants ont révélé l'importance de la dimension anxieuse chez ce patient. Outre sa réticence à prendre des psychotropes prescrits, il n'hésitait pas à lire les prospectus, rapportant des effets secondaires avec une fréquence très exagérée. L'alliance thérapeutique était très délicate, et ne fut maintenue que grâce à la batterie d'examens paracliniques que l’équipe soignante lui a fait faire à la recherche d'une étiologie organique à son trouble.

Le bilan neurocognitif et les explorations biologiques multiples, dont une biopsie cutanée, n'ont pas révélé d'anomalies notables en dehors de calcifications bilatérales non spécifiques des noyaux gris centraux à l'IRM cérébrale. Au terme de ces investigations exhaustives, le diagnostic d'une hypochondrie ou d'un trouble délirant type somatique ont été discutés. La conviction délirante inébranlable nous a fait retenir le diagnostic d'un syndrome d'Ekbom primaire, correspondant au diagnostic DSM IV-TR au trouble délirant type somatique à thématique d'infestation parasitaire, compliqué d'un épisode dépressif caractérisé. Après une tentative non concluante de traitement par Olanzapine pendant 6 semaines (10mg/ jour), une atténuation significative du délire a été obtenue sous Halopéridol (12mg /j) au bout d'un mois de traitement. Par ailleurs, l’épisode dépressif a été jugulé par un traitement à base de Venlafaxine (75mg / jour).

### Observation n° 2

Le deuxième patient a été diagnostiqué et référé par un service de dermatologie pour prise en charge. C'est un célibataire âgé de 47 ans, de niveau scolaire correspondant au baccalauréat qui travaille comme électricien. Il est sans antécédents pathologiques particuliers, en dehors de crises de migraines sans aura intermittentes depuis l’âge de 20 ans sans traitement suivi. Il se plaint depuis 18 ans d'un prurit avec des sensations de reptations sous sa peau qu'il impute à des parasites. L'anamnèse détaillée avec le patient révèle le caractère évolutif irrégulier de ce trouble. En effet le patient rapporte quelques périodes « d'accalmie » qui durent quelques semaines, durant lesquelles les symptômes semblent s'atténuer spontanément avant de reprendre ensuite durant plusieurs mois. La revue de l'historique du patient objective aussi l'existence de deux périodes d'arrêt de toute activité professionnelle concomitantes à des symptômes du registre dépressif (un désintérêt pour le travail et les loisirs, une tristesse associée à une fatigue quasi-permanente avec une anorexie et une insomnie). Le premier épisode est survenu à l’âge de 27 ans et coïncidait avec le début de la symptomatologie en rapport avec le délire d'infestation parasitaire. Le tableau thymique s’était alors spontanément résolu au bout de quelques mois. Cependant les sensations sous-cutanées désagréables avaient persistés longtemps après. Le deuxième épisode dépressif date de 8 ans, et parait similaire en tout point en premier. En outre, la recherche dans les antécédents de symptômes appartenant au registre maniaque ou hypomaniaque demeure infructueuse. Globalement, et en dehors du premier épisode, la « symptomatologie cutanée » semble évoluer indépendamment des épisodes thymiques puisque le patient insiste sur sa survenue de façon isolée.

Par ailleurs, l'interrogatoire révèle l'existence d'une symptomatologie somatique subjective variable et dispersée dans le temps. Ainsi, on a noté l'existence d’épisodes récurrents et asynchrones de fourmillement et de faiblesse transitoire affectant les membres supérieurs, préférentiellement le droit. Ces symptômes ont débuté vers l’âge de 30 ans et surviennent de façon espacée de quelques mois. La résolution est spontanée en quelques heures, plus rarement en quelques jours. Le patient semble incapable de rapporter le nombre de ces épisodes avec précision et semble en minimiser l'ampleur. De plus le patient a présenté il y'a 8 mois par un épisode transitoire d’à peine quelques heures, durant lequel son discours est devenu totalement incompréhensible. Cet épisode, constaté par l'entourage du patient, est très évocateur d'une aphasie de compréhension spontanément réversible. L'exploration du contenu ne relève pas d'idées délirantes autres que le délire d'infestation parasitaire. On ne note aucune autre idée de nature hypochondriaque ou appartenant au registre obsessionnel ou dépressif. L'idée d'infestation par le parasite, dont la conviction est inébranlable, paraît étayée par des troubles perceptifs correspondant à des hallucinations tactiles « ascendantes » au niveau de la région génitale, le tronc et le cou. Le patient les décrit comme un mouvement de reptation perçu, accompagné d'un prurit intense qu'il impute aux morsures du parasite qui déchire sa peau pour se frayer chemin. Il affirme même pouvoir arrêter la progression sous-cutanée de ces « bestioles » en interposant son doigt sur leurs trajectoires. Malgré des tentatives d'extraction répétées, le patient dit ne jamais avoir pu voir le parasite. Le reste de l’évaluation psychiatrique semblait normal en dehors de plaintes concernant des difficultés de concentration au travail d'apparition récentes et une insomnie d'endormissement.

**Figure 2:**
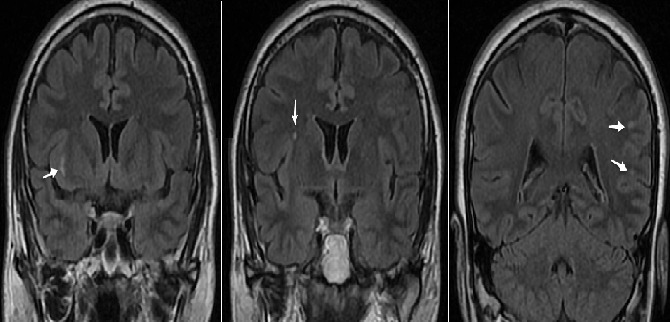
IRM cérébrale en séquence FLAIR chez le 2ème patient: Les coupes coronales montrent quelques lésions nodulaires en hypersignal FLAIR intéressant la substance blanche sous corticale fronto-pariétale bilatérale, temporale et capsulaire externe droite en rapport avec des infarctus lacunaires sous corticaux. La topographie des anomalies de signal prédominant dans les territoires péri-ventriculaires et les centres semi-ovales est très évocatrice du CADASIL

L'examen somatique a objectivé de graves lésions dermatologiques occasionnées par l'application répétée de détergents et d'insecticides sur son appareil génital pour « éradiquer le nids de ces parasites ». Le bilan neuropsychiatrique a objectivé une légère altération du MMS (à 25) et une augmentation du temps de traitement au Test de Stroop en faveur d'une atteinte des fonctions exécutives. Le reste de l'examen, notamment neurologique, n'avait pas objectivé d'anomalies notables. Un bilan biologique et radiologique a été demandé. Les coupes coronales en séquence FLAIR de l'IRM ont montré quelques lésions nodulaires en hypersignal intéressant la substance blanche sous-corticale fronto-pariétale bilatérale, temporale et capsulaire externe droite en rapport avec des infarctus lacunaires sous corticaux (Figure 2). La nature et topographie de ces lésions étaient fortement évocatrices d'une démence vasculaire sous-corticale consécutive à une leuco-encéphalopathie type CADASIL. La confirmation par biologie moléculaire étant indisponible, le diagnostic provisoire d'un syndrome d'Ekbom secondaire à une pathologie organique, en l'occurrence une maladie des petites artères cérébrale, a été retenue. Une prescription d'Halopéridol à 6mg / jour a été faite avec une évolution favorable et une rémission totale au bout de 7 semaines de traitement. Le patient est depuis suivi en consultation de neurologie.

### Observation n° 3

**Figure 3:**
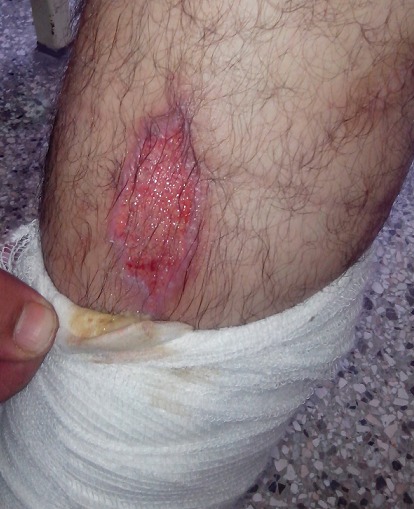
Ulcération pré-tibiale droite auto-infligée par le 3ème patient dans une tentative d'extraire le parasite. On peut remarquer plus haut des hyperpigmentations correspondant à des ulcérations anciennes cicatrisées

Le troisième patient a été vu en milieu carcéral. La consultation psychiatrique a été motivée par une demande du dermatologue qui a suspecté des lésions auto-infligées, prenant la forme d'ulcérations plus au moins profondes et larges au niveau des membres inferieurs, supérieurs et de l'abdomen (Figure 3). Le patient, âgé de 38 ans, est un père de famille qui travaille comme menuisier. Il est détenu depuis 18 mois pour usage et trafic de cannabis. La revue de ses antécédents a objectivé une consommation régulière de cette substance psychoactive depuis une vingtaine d'année, avec un sevrage forcée durant la période de son emprisonnement. Il n'y avait par ailleurs pas d'autres antécédents personnels ou familiaux notables. L'anamnèse avec le patient révèle que ces lésions sont la conséquence du grattage réactionnel à un prurit intense. Ce prurit serait induit par « des vers qui colonisent la peau du patient ». Selon ce dernier, l'infestation serait survenue à peine trois mois après son incarcération et épargnerait bizarrement ses compagnons de cellule. Malgré une hygiène rigoureuse consistant en des lavages répétés et une multitude de traitements dermatologiques, l’état du patient ne s'est pas amélioré. Cliniquement, le patient était de bon contact, et ne présentait ni signes de désorganisation patents ni bizarrerie comportementale. Son état émotionnel était dominé par la tristesse et l'angoisse générée par cette « infestation » et surtout la peur de contaminer les membres de sa famille, compte tenu de sa proche libération. La pensée du patient était globalement cohérente et demeurait centrée sur l'infestation. Il décrivait avec détail les trajets des vers sous sa peau et exprimait son désespoir face à l'inefficacité de tous les traitements qu'il avait pris. Nous n'avions pas noté d'autres thématiques délirantes, en dehors des quelques idées de référence étroitement liée à l'idée de l'infestation: « j'ai la vague impression que les autres détenus m’évitent au maximum. Par exemple, ils ont l'air très gênés en me serrant la main et certains d'entre eux se lavent aussitôt de peur d’être contaminés.» disait-il. Sur le plan perceptifs et outres les hallucinations tactiles à l'origine de l'idée d'infestation, le patient se plaignait d'hallucinations auditives « j'entends souvent des voix étrangères qui parlent de moi et surtout de mon hygiène sur un ton moqueur». Il rapporte que ces voix s'adressent parfois directement à lui pour l'insulter ou lui ordonner d'aller se laver. Du reste, le patient ne présentait pas de déficience cognitive patente et ses fonctions instinctuelles n’étaient pas perturbées. La normalité des bilans pratiqués et la richesse du tableau hallucinatoire, ainsi que la thématique délirant nous ont fait discuté un épisode dépressif majeur avec des caractéristiques psychotiques ou une schizophrénie. Le patient a favorablement évolué sous Risperidone 4mg /jour en association avec la fluoxétine 20mg /j. Une amélioration significative de l'humeur a été obtenue au bout de 5 semaines et les idées délirantes ont régressé terme de 7 semaines de traitement.

## Discussion

### Epidémiologie

D'un point de vue épidémiologique, le syndrome d'Ekbom est une affection rare qui touche préférentiellement les femmes avec un sex-ratio autour de 2,8 [4]. L’âge de début est généralement avancé (64 ans en moyenne) et la durée d’évolution moyenne avant le premier contact avec le psychiatre est de trois ans [1]. La fréquence élevée de l'isolement social [1, 2] ou de la personnalité paranoïaque [1] ont été relevés comme traits prémorbide par certains auteurs. Ces données contraste avec le profil épidémiologique de nos trois malades, tous de sexe masculin avec un âge de début du trouble plutôt jeune. Alors que la plupart des auteurs décrivent une évolution d'un seul tenant, la possibilité d'une évolution périodique a été évoquée par Skott dans [4]. Ce dernier mode évolutif a été également observé chez notre 2^ème^ patient qui reste par ailleurs le seul des trois à vivre la situation d'isolement social souvent rapportée dans les descriptions de ce syndrome [1, 2, 4].

### Nosographie et diagnostic différentiel

Si les nosographies actuelles s'accordent à placer les délires monothématiques sous-tendus par des hallucinations de l'organe cutané dans la rubrique des Troubles délirants [5, 6], l'exercice diagnostique devant un patient se plaignant d'infestation parasitaire demeure long et difficile. En effet, une vraie ectoparasitose ne doit jamais être écartée sans avoir conduit une anamnèse détaillée et des investigations dermatologiques poussées. Un trouble factice, une Hypochondrie ou un Trouble Obsessionnel Compulsif (avec des obsessions de contaminations) voire une simulation restent également des diagnostics envisageables selon le contexte clinique et paraclinique du patient.

### Cadres diagnostiques

D'un point de vue étiologique, il faudra nuancer entre le délire d'infestation parasitaire primaire et secondaire [2, 3, 7]. Dans la forme primaire, individualisée par Ekbom, les hallucinations sont limitées à la peau et les idées délirantes sont monothématiques. Cette description correspond parfaitement à notre premier patient, chez qui le bilan étiologique s'est avéré négatif. Dans la forme secondaire du délire, les hallucinations cutanées et la conviction délirante de l'infestation peuvent prendre place dans un ensemble délirant plus large, imputable à une pathologie psychiatrique ou organique [2, 3, 7]. Cette forme serait la plus fréquente selon certains auteurs [2]. De ce fait, des affections telles que l'insuffisance hépatique ou rénale, l'ictère, l'anémie, les carences vitaminiques ainsi que certaines infections virales (VIH, Hépatites) sont essentielles à considérer, car elles peuvent se manifester par un prurit ou des fourmillements et représentent des étiologies sous-jacentes potentiellement traitables [3, 7]. Une cause organique cérébrale doit aussi être impérativement écartée: les démences, les tumeurs, les infarctus cérébraux et les méningo-encéphalites [7, 8] peuvent se manifester par un syndrome d'Ekbom nécessitant des investigations spécifiques orientées en fonction du contexte clinique.

Ainsi, l'IRM cérébrale de notre deuxième patient a mis en évidence des lésions très évocatrice du CADASIL (Cerebral Autosomal Dominant Arteriopathy with Subcortical Infarcts and Leukoencephalopathy). Ce terme a été proposé en 1993 par T*ournier-Lasserve* et *Bousser* pour désigner une rare affection héréditaire autosomique dominante (gène NOTCH 3 sur le chromosome 19) touchant les petites artères cérébrales et responsables d′infarctus sous-corticaux et d′une atteinte de la substance blanche [9]. Malgré des variations interindividuelles et interfamiliales importantes de la présentation clinique, l'histoire naturelle de cette maladie est caractérisée par l′apparition de crises de migraine avec aura au cours de la 2^ème^ ou 3^ème^ décennie, suivies une dizaine d′années plus tard par des accidents ischémiques cérébraux récidivants (transitoires ou constitués) et après environ 20 ans d′évolution, par une démence (80% des cas) et une hémi- ou tétraparésie avec perte totale de l'autonomie (90% des patients) [9]. Ce tableau s'accompagne souvent d'atteintes cognitives dont la plus fréquente reste l'altération des fonctions exécutives qui apparaît précocement chez les jeunes patients paucisymptomatiques. Les troubles de l'attention et de la mémoire de type sous-cortico-frontal n'apparaissent que plus tard au cours de l’évolution [10]. Le cours de la maladie est aussi émaillé d’épisodes thymiques maniaques ou dépressifs dans 20% des cas [9], alors la comitialité est peu observée (5 à 10% des cas) [11]. Le décès survient habituellement durant la sixième décade, souvent à cause des complications pulmonaires liées à des troubles de la déglutition [9].

L′imagerie par résonance magnétique (IRM) est essentielle au diagnostic du CADASIL. Les anomalies de signal en IRM peuvent être détectées durant la phase présymptomatique de la maladie et notamment dès l′âge de 20 ans [9]. Après 35 ans, tous les sujets porteurs du gène muté, mêmes asymptomatiques, ont une IRM anormale [9]. Les lésions typiques observées chez ces patients sont les hypersignaux de la substance blanche, les petits infarctus sous-corticaux, et les microsaignements. La topographie lésionnelle périventriculaire et sous corticale au niveau de la partie antérieure des lobes temporaux et/ou des capsules externes rendent encore plus plausible le diagnostic [9]. C'est cette topographie lésionnelle à l'IRM qui a orienté le diagnostic chez notre 2^ème^ patient, qui par ailleurs, présente un profil évolutif et cognitif correspondant parfaitement au descriptions du CADASIL (crises de Migraine, accidents ischémiques transitoires à répétition, des épisodes thymiques récidivants, des troubles des fonctions exécutives et de l'attention et des antécédents familiaux d'accidents vasculaires cérébraux). Cependant, notre revue de la littérature n'a pu retrouver de cas rapportés de syndrome d'Ekbom chez des patients souffrant du CADASIL, les seuls troubles psychiatriques y étant classiquement associés, sont les troubles de l'humeur (maniaques ou dépressifs) [9]. Enfin, le diagnostic de certitude de CADASIL chez notre patient n'a pu être fait, du fait de l'indisponibilité du test moléculaire qui met en évidence la mutation caractéristique au sein du gène NOTCH 3. Dans sa forme secondaire à une pathologie psychiatrique, le syndrome d'Ekbom peut relever d'un état hallucinatoire induit directement par une substance psychotrope. Les descriptions les plus fréquentes sont associées à un usage de cocaïne, de méthamphétamine, de chloral ou dans le cadre d'un sevrage alcoolique (Délirium trémens avec zoopsies) [1, 3, 4, 7, 12]. Des cas survenus sous une médication par corticostéroïdes ont été également observés [3, 4, 7, 12].

L'idée de l'infestation peut être faire aussi partie du tableau clinique d'une schizophrénie ou d'un autre trouble psychotique [1, 3, 4, 7, 12]. Dan ce cas, le polymorphisme délirant et la présence d’éléments de désorganisation et/ou de symptômes négatifs permettraient d'orienter le diagnostic. Lorsque le délire d'infestation, vécu comme un châtiment mérité, est accompagné d'une tristesse de l'humeur et d'une peur culpabilisante d’être contagieux et de nuire aux proches, le diagnostic d'un épisode dépressif avec des caractéristiques psychotiques doit être évoqué en premier. Ce cadre nosographique serait l'un des plus fréquents après le trouble délirant primaire, selon certains auteurs. [4]. Ces deux derniers diagnostics ont été longuement discutés chez notre 3^ème^patient, chez qui le bilan étiologique organique s'est par ailleurs révélé négatif. Outre le délire d'infestation à mécanisme hallucinatoire tactile et cénesthésique, le patient présentait des hallucinations acoustico-verbale impératives et insultantes très angoissantes tournant autour de la thématique de l'infestation. Le désespoir, le souci excessif de contaminer les membres de sa famille une fois remis en liberté ainsi que le sentiment d’être sale et rejeté par les autres compagnons de cellule étaient très évocateurs d'un état dépressif délirant. L'absence de bizarrerie ou de symptômes de désorganisations confortait cette hypothèse diagnostique.

### Traitement

La prise en charge du syndrome d'Ekbom s'avère être la plupart du temps une tache difficile. La résistance à la « psychiatrisation » de leurs symptômes entrainent les patients dans une spirale de surconsommation de soins somatiques [13] et la frustration occasionnée peut être à l'origine de comportements suicidaires ou dangereux pour l'entourage [13]. Les cas retrouvé dans la littérature qui font état de patients traités avec succès par électro-convulsivothérapie [3] ou antidépresseurs [13, 14] sont anecdotiques et la psychothérapie ne semble occuper qu'une place de traitement adjuvant dans cette indication [4, 13]. Dès leur découverte, les neuroleptiques de première génération se sont imposés comme le traitement de choix des patients souffrant du délire d'infestation. Parmi ces molécules, le Pimozide se démarquent nettement du lot, étant le seul neuroleptique ayant démontré son efficacité dans un essai clinique en double aveugle type « cross over » [15]. W. Trabert note aussi l'efficacité supérieure du Pimozide dans un large revue de la littérature portant sur 1223 cas rapportés [2]. Dans une série de publications récentes sur la place des neuroleptiques dans le délire d'infestation parasitaire, Lepping et Freudnmann ont retenu 63 cas rétrospectifs sur 434 publications recensées jusqu'en 2007, dont 154 depuis l'introduction des antipsychotiques de seconde génération [3, 16]. Leur bilan reste assez mitigé. Outre le fait de rappeler que les prescriptions des neuroleptiques atypiques dans le délire d'infestation parasitaire se font hors AMM, ils soulignent qu'aucun neuroleptique de 1^ère^ ou de 2^ème^ génération n'a démontré un niveau de preuve élevé dans l'indication du délire d'infestation [3]. Les deux auteurs ne recommandent plus le Pimozide comme 1^er^ choix, pour des raisons d'innocuité [16] et lui préfèrent les molécules de seconde génération tel la Risperidone, l'Olanzapine et l'Amisulpride [16].

## Conclusion

Face à un délire d'infestation, le praticien se heurte à trois niveaux de difficultés. D'abord le caractère trans-nosographique de ce syndrome, qui complique la démarche diagnostique. Ensuite, il y'a la difficulté d’établir une alliance thérapeutique avec le patient, vu sa résistance à la « psychiatrisation » du symptôme qui l'amène à consulter. Enfin, le vide en matière de recommandations thérapeutiques, qui laisse le médecin livré à lui même face à un patient « difficile » à gérer. On ne peut donc que souligner la nécessité urgente d’établir une démarche diagnostique codifiée et des recommandation thérapeutiques basés sur les preuves, afin d'assister le praticien dans sa tache complexe, parfaitement illustrée par nos trois observations cliniques.
